# Mapping of the Spinal Sensorimotor Network by Transvertebral and Transcutaneous Spinal Cord Stimulation

**DOI:** 10.3389/fnsys.2020.555593

**Published:** 2020-10-09

**Authors:** Polina Shkorbatova, Vsevolod Lyakhovetskii, Natalia Pavlova, Alexander Popov, Elena Bazhenova, Daria Kalinina, Oleg Gorskii, Pavel Musienko

**Affiliations:** ^1^Institute of Translational Biomedicine, Saint Petersburg State University, Saint Petersburg, Russia; ^2^Pavlov Institute of Physiology Russian Academy of Sciences, Saint Petersburg, Russia; ^3^Russian Research Center of Radiology and Surgical Technologies, Ministry of Health of the Russian Federation, Saint Petersburg, Russia; ^4^Children’s Surgery and Orthopedic Clinic, Department of Non-pulmonary Tuberculosis, Institute of Phthysiopulmonology, Saint Petersburg, Russia

**Keywords:** transvertebral spinal cord stimulation, transcutaneous stimulation, sensorimotor network, spinal cord, decerebrated rat, neuromodulation

## Abstract

Transcutaneous stimulation is a neuromodulation method that is efficiently used for recovery after spinal cord injury and other disorders that are accompanied by motor and sensory deficits. Multiple aspects of transcutaneous stimulation optimization still require testing in animal experiments including the use of pharmacological agents, spinal lesions, cell recording, etc. This need initially motivated us to develop a new approach of transvertebral spinal cord stimulation (SCS) and to test its feasibility in acute and chronic experiments on rats. The aims of the current work were to study the selectivity of muscle activation over the lower thoracic and lumbosacral spinal cord when the stimulating electrode was located intravertebrally and to compare its effectiveness to that of the clinically used transcutaneous stimulation. In decerebrated rats, electromyographic activity was recorded in the muscles of the back (m. longissimus dorsi), tail (m. abductor caudae dorsalis), and hindlimb (mm. iliacus, adductor magnus, vastus lateralis, semitendinosus, tibialis anterior, gastrocnemius medialis, soleus, and flexor hallucis longus) during SCS with an electrode placed alternately in one of the spinous processes of the VT12–VS1 vertebrae. The recruitment curves for motor and sensory components of the evoked potentials (separated from each other by means of double-pulse stimulation) were plotted for each muscle; their slopes characterized the effectiveness of the muscle activation. The electrophysiological mapping demonstrated that transvertebral SCS has specific effects to the rostrocaudally distributed sensorimotor network of the lower thoracic and lumbosacral cord, mainly by stimulation of the roots that carry the sensory and motor spinal pathways. These effects were compared in the same animals when mapping was performed by transcutaneous stimulation, and similar distribution of muscle activity and underlying neuroanatomical mechanisms were found. The experiments on chronic rats validated the feasibility of the proposed stimulation approach of transvertebral SCS for further studies.

## New and Noteworthy

Neuromodulation of the sensorimotor network distributed rostrocaudally over the lumbar and sacral spinal segments by transvertebral electrical stimulation.

## Introduction

Spinal cord stimulation (SCS) is an effective method of recovery after spinal cord injury (SCI) and other disorders that are accompanied by motor and sensory deficits (Shapkova, 2004; Harkema et al., 2011; Zhong et al., 2019). Several approaches can be taken for electrode setting near the spinal cord: subdural (e.g., Minev et al., 2015; Capogrosso et al., 2018a), epidural (e.g., Musienko et al., 2005, 2009; Lavrov et al., 2006), transcutaneous (e.g., Minassian et al., 2007; Roy et al., 2012; Sayenko et al., 2015; Hofstoetter et al., 2018), or subcutaneous (Pavlova et al., 2019). In chronic studies (Schmidt et al., 1978) and intraoperative monitoring (e.g., Calancie et al., 1994), the target of stimulation may not be the spinal cord itself but a selective group of dorsal or ventral roots. Depending on the degree of invasiveness, the stimulation through electrodes located above the spinal cord can cause a certain selectivity of motor neuron pool activation, and this changes the effectiveness of the technique.

The least invasive transcutaneous SCS is now frequently used in studies on healthy humans aimed at central pattern generator research and central and peripheral neuronal control of locomotor activity (Gerasimenko et al., 2014, 2016; Gerasimenko Y. et al., 2015). This method has been efficiently applied and widely used in a clinical practice for neurorehabilitation of patients with severe SCI (Gerasimenko Y.P. et al., 2015; Gad et al., 2018a,b). However, further development of the method of transcutaneous stimulation requires suitable animal models. Unfortunately, in animals, the practical tasks of stable electrode fixation on the hairy and easily movable skin surface during longitudinal studies, as well as achieving identical electrode placement in several animals, are complicated and require considerable skill and patience (Peckham and Knutson, 2005). The transcutaneously induced locomotion in cats is more unstable and less coordinated than the epidurally induced locomotion, presumably due to instability of the electrode position (Musienko et al., 2013). Therefore, the method of non-invasive SCS is rarely used in these experiments although its effectiveness has been shown for recruiting spinal sensorimotor pathways and for initiation of the locomotor activity in acute decerebrate (Musienko et al., 2013) and chronic spinal cats (Edgerton et al., 2013; Musienko et al., 2013). In the current work, we have proposed a suitable approach for electrode implantation into the vertebral spinous processes for transvertebral SCS. This method has allowed us to quickly and stably fix the electrodes relative to the vertebral column and spinal cord segments in accordance with the skeletotopy relationships (Wenger et al., 2016; Shkorbatova et al., 2019). However, chronic transvertebral stimulation is still an experimental technique with significant differences compared to clinical protocols.

The neuronal mechanisms of either strongly invasive or less invasive SCS are not well defined, although some aspects have been investigated and discussed in a number of experimental papers (Gerasimenko et al., 2003; Musienko et al., 2005, 2012; Lavrov et al., 2006; Capogrosso et al., 2013). The consensus view is that the SCS effects are based on the recruiting of sensory inputs of the dorsal cord and roots lying under the stimulating electrodes, followed by polysynaptic activation of the sensorimotor neuronal circuits (Musienko et al., 2012). This underlying mechanism and anatomical spreading of the afferent fibers carrying the sensory input to the spinal network allowed the use of epidural SCS in a spatiotemporal neuromodulation mode that significantly improved the positive effects of stimulation after spinal sectioning (Musienko et al., 2009; Wenger et al., 2014, 2016). One of the important unanswered questions is whether it is possible to effectively recruit by surface SCS the specific neuronal pathways widely distributed rostrocaudal over the lumbar and sacral spinal segments (Merkulyeva et al., 2018).

We performed electrophysiological mapping of the spinal sensorimotor pathways by low thoracic and lumbosacral transvertebral SCS and recording of the activity of the multiple hindlimb and trunk muscles participating in normal locomotion and postural tasks.

The data obtained have shown that the muscle responses to stimulation are topical and reflect the rostrocaudal distribution of the corresponding motor neuron pools in the lumbosacral spinal cord. The received distribution of muscle activity was compared with the mapping performed by transcutaneous stimulation. The validity of the proposed stimulation approach was studied in a chronic experiment.

## Materials and Methods

### Subjects

The study was performed on adult male Wistar rats (300–350 g body weight). All experimental procedures were approved by the Ethics Commission of the Pavlov Institute of Physiology. Experiments were performed in full accordance with the requirements of Council Directive 2010/63EU of the European Parliament on the protection of animals used for experimental and other scientific purposes. Before the experiments, the rats were housed with two to three animals per cage with free access to food and water. Eight rats were used for transvertebral mapping, and six rats were used for comparative study of transvertebral vs. subcutaneous mapping. Six rats were used in the chronic experiment to check the stability of muscle responses to transvertebral stimulation.

All surgical procedures were conducted under isoflurane anesthesia (4% for induction, 1–2% for maintenance, mixed with oxygen, flow rate 0.8 L/min). During surgery, the animals were placed on a heating pad at a temperature of 37°C and received injections of 2 ml of warm 0.9% NaCl subcutaneously every 2 h to prevent dehydration.

### EMG Implantation

To record the EMGs, stainless steel wire electrodes (AS632, Cooner Wire, Chatsworth, CA, United States) were prepared by removing a small notch of insulation (0.5 mm) on each wire to expose the conductor. The wires were inserted into the muscle through a 23G needle and positioned in the middle of the muscle in the most responsive part, which was identified by electrical stimulation (“hot spot”), then the wires were fixed together with Ethilon 4 suture at the entrance and exit from the muscle (Capogrosso et al., 2018b). The EMG signals were differentially amplified (A-M Systems United States, model 1700, bandwidth of 10 Hz–5 kHz) and digitized at 20 kHz with a National Instrument A/D board.

### Muscle Set

In the acute experiment, the electrodes were implanted into the muscles of the back [m. longissimus dorsi (LD) near the VT13 vertebra], tail [m. abductor caudae dorsalis (ACD)], and hindlimb [m. iliacus (IL), m. adductor magnus (ADD), m. vastus lateralis (VL), m. semitendinosus (ST), m. tibialis anterior (TA), m. gastrocnemius medialis (GM), m. soleus (SOL), and m. flexor hallucis longus (FHL)]. The selected muscles had to meet at least one of three criteria. The first was that the muscles are widely used in studying locomotion (e.g., IL, VL, ST, GM, TA) or postural control (e.g., LD, ACD, SOL, ADD) (Siegel, 1970; Karayannidou et al., 2009; Musienko et al., 2014). The second was that they are the hindlimb muscles having the most rostral (ADD, IL) or the most caudal motoneuronal pools (FHL) in the spinal cord (Mohan et al., 2015; Wenger et al., 2016). The third was that they served as reference points with the most rostral (LD) or the most caudal (ACD) motoneuronal pools in our zone of interest.

For chronic muscle implantation, the TA and GM of one leg were chosen because they are most often used in chronic experiments as antagonist muscles to analyze a gait pattern.

### Transcutaneous Mapping

The transcutaneous stimulation was conducted using a 5 × 5-mm electrode made of biocompatible self-adhesive conductive hydrogel (FDA 510(k) Premarket Notification K092546; CWN2505; GMDASZ Manufacturing Co., Ltd., Shenzhen, China) placed between the spinous processes of the VT12/VT13, VL1/VL2, and VS1/VS2 vertebrae. The vertebrae were carefully palpated and the VL6/VS1 vertebral junction was determined as the last movable joint before four sacral vertebrae, which were immobile relative to each other. The skin under the intervertebral spaces was then marked, but the exact position was checked every time before electrode placement. The 30 × 50-mm ground electrode (made of the same material) was fixed on the animal’s abdomen.

### Transvertebral Stimulation

**FIGURE 1:**
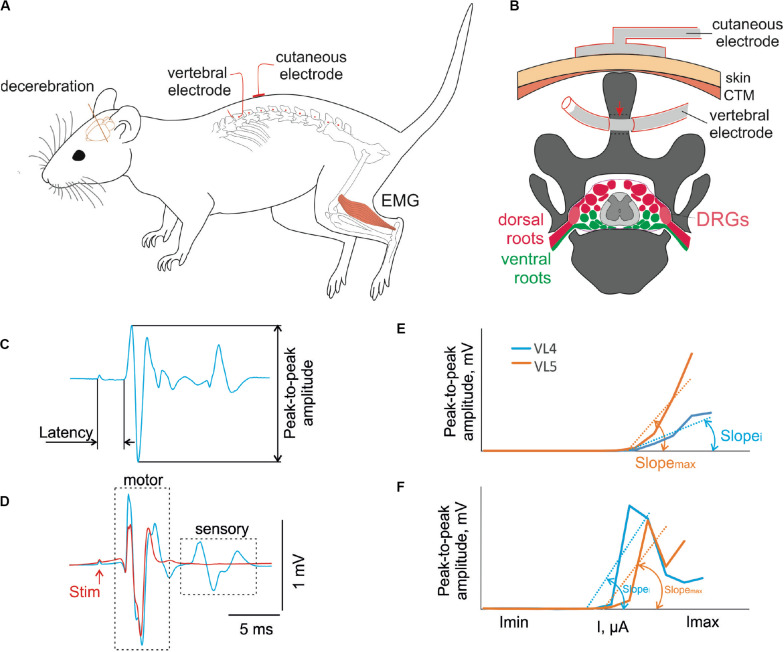
**(A)** Design of the experiment. The decerebrate rat is fixed in the custom stereotaxic frame and the holes were drilled in the spinous processes of the VT12–VS1 (red dots) vertebrae. The stimulating vertebral electrode was placed alternately in the hole in each vertebra. The stimulating cutaneous electrode was placed in the zones between adjacent vertebrae. The intramuscular electrodes were implanted to record EMG signals. **(B)** The representative scheme of the frontal section of vertebra VL2 with the spinal cord, dorsal (red) and ventral (green) roots, and dorsal root ganglia (DRGs, pink) inside the vertebral canal. The position of the stimulating cutaneous electrode and vertebral electrode in the spinous process is shown. The contact insulation-free area of the vertebral electrode indicated with a red arrow. **(C)** An example of motor-evoked potential with the main characteristics measured. **(D)** An example of motor-evoked potential to first (blue) and second (red) pulse of double-pulse stimulation. **(E,F)** The recruitment curves of motor **(E)** and sensory **(F)** responses at stimulation of different vertebrae and their slopes (max, maximal slope; *i*, slope at stimulation of some other vertebra).

Transvertebral mapping of the lower thoracic, lumbar, and sacral spinal segments was conducted in acute experiments after cutting the skin and the fascia on the back to expose the spinous processes of the vertebrae VT11–VS2 and separate them from the surrounding tissues. A hole (1 mm diameter) was drilled with a hand drill horizontally (Figure 1B) into each spinous process of vertebrae VT12–VS1 close to its basement.

For acute experiments, the wire electrode (2 mm of the Teflon insulation was removed around the wire, 2 cm from the wire tip) was placed alternately in one of the spinous processes of the VT12–VS1 vertebrae and fixed in the entrance and exit of the spinous process canal. For chronic experiments, the spinous process of the L2 vertebra was exposed through a minimal skin and muscle incision and drilled horizontally. The vertebral stimulation electrode was fixed inside the hole by tying a knot around the dorsal part of the spinous process. The skin was then closed with Ethilon 4. Two common ground (indifferent EMG and stimulation grounds) wires (with 1 cm of the Teflon removed distally) were inserted into the muscles near the right and left shoulders. The scheme of the stimulating electrode position in the VL2 spinous process is presented in Figure 1B.

For acute experiments after bilateral carotid artery ligation, the animal was decerebrated at the precollicular–postmammilar level (Dobson and Harris, 2012) and placed into a custom stereotaxic frame, where it was fixed with vertebral clamps for subsequent recordings as shown in Figure 1A. The head and the tail were supported with stripes of soft fabric. The hindlimbs were in the unsupported state. The anesthesia was turned off just after the decerebration.

For chronic animals, the recording electrodes were implanted into the muscles of one leg as described above and the wound was closed using Vicryl 5 for the fascia and Ethilon 4 for the skin. All wires were coiled in the back region to form a stress release loop and were combined into an Amphenol head connector, which was fixed on the animal’s head. After surgery, the animal was let to recover from anesthesia in a warm box. Analgesic (ketorolac, 1 mg/kg, s.c.) and antibiotic (enrofloxacin, 5 mg/kg, s.c.) were administered during 3 and 5 days after surgery, respectively.

### Electrical Stimulation and Recording

The recruiting was performed by stimulation with single pulses of 1 Hz frequency at stimulation intensities ranging from 1,500 to 4,500 μA for transcutaneous stimulation and from 500 to 3,300 μA for transvertebral stimulation in increments of 100 μA, with a pulse duration of 0.2 ms, interstimulus interval of 1 s, and 10 impulses for each current (example of response is in Figure 1C). Separation of the motor and sensory components of the muscle response was achieved using stimulation by double impulses with interpulse interval of 20 ms (Gerasimenko et al., 2006; Roy et al., 2012). Preliminary experiments confirmed that with submaximal currents the sensory components of the muscle response to the second part of a double impulse were suppressed (Figure 1D). However, in some cases, they reappeared in further current increases as described previously (Minassian et al., 2004). This is why the recruiting by itself was performed with a single impulse stimulation. The values of the latencies received by double-impulse stimulation were used to separate the early response component from the others during the single impulse stimulation.

### Anatomical Verification of Spinal Pathways Under the Stimulating Electrodes

At the end of the experiment, the animal was perfused with 100 ml of 0.9% NaCl followed by 350 ml of 4% paraformaldehyde in 0.1 M phosphate buffered saline (PBS). A careful dissection was then performed to explore the lengths and the positions of the sT12–S1 spinal segments, the dorsal root ganglia (DRG) and the dorsal and ventral roots in relation to the VT12–VS1 vertebrae. The mean skeletotopy of the spinal segments was plotted based on these data.

### Analysis and Statistics

Custom scripts written in MATLAB were used to measure the evoked potentials from the selected muscles. We analyzed the latency and peak-to-peak amplitude of the different response components in the 0.5 to 15 ms range after the stimulation impulse (Figure 1C). The “early” response (ER), which did not deteriorate during the double stimulation test (Gerasimenko et al., 2006) at submaximal current (Figure 1D), had the minimal latency. We attributed this to the motor response due to the direct activation of the motoneuronal axons. The “medium” response (MR) was second in terms of latency, just after the ER. The “late” response (LR) was third in terms of latency, just after the ER and MR. Both the MR and LR were deteriorated during the double stimulation test (Gerasimenko et al., 2006) as reflex (sensory) responses required time for recovery for synaptic transmission.

The recruitment curves for motor and sensory responses were constructed based on these values. For each muscle of each animal, the distribution of the recruitment curves was received over a range of stimulated vertebra. The slopes of the ascending parts of these curves were calculated by the least squares method (e.g., Figures 1E,F, Slope*_i_* and Slope_max_), where the beginning value had to be greater than a 0.1-mV threshold. All slopes of the recruitment curve distributions were normalized to the maximal slope value (Figures 1E,F, Slope_max_). For transvertebral mapping, *i* = (VT12–VS1); for comparison of the transvertebral stimulation with the transcutaneous one, *i* = VT12, VL2, VL6.

The distributions of the normalized slopes of the recruitment curves of one muscle were averaged over all animals. The χ^2^ two-sample Bonferroni-adjusted test (Press et al., 1992) was used when comparing these distributions for different muscles. The paired Wilcoxon criteria was applied to compare the normalized slopes of different muscles in one stimulation point when comparing transvertebral and transcutaneous stimulation. For each muscle, the latencies and threshold currents of the ERs for the recruitment curve that had maximal slope were averaged over animals. The Friedman test with *post hoc* Bonferroni adjustment was used to compare these values for the different muscles.

All data are reported as mean ± SE. The criterion level for the determination of statistical difference was set at *p* < 0.05.

## Results

### Transvertebral Spinal Cord Stimulation

**FIGURE 2:**
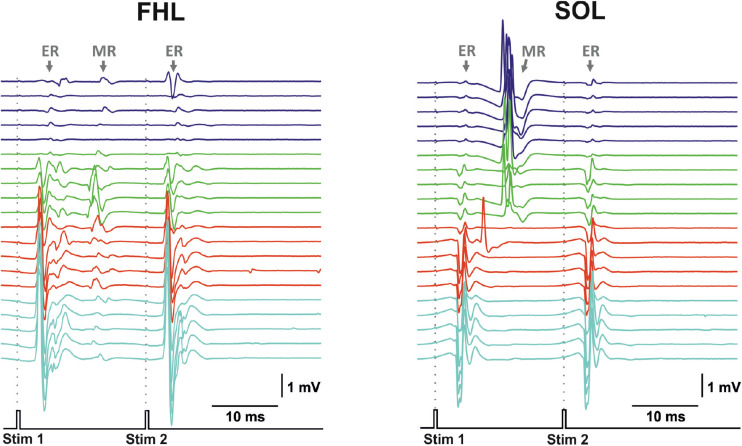
The representative examples of evoked potential dynamics with increasing current for transvertebral double-pulse electrical stimulation (1,900–2,200 μA) delivered at VL5 vertebrae for mm. flexor hallucis longus (FHL) and soleus (SOL). Stim, stimulation impulse; ER, early response; MR, medium response.

**FIGURE 3:**
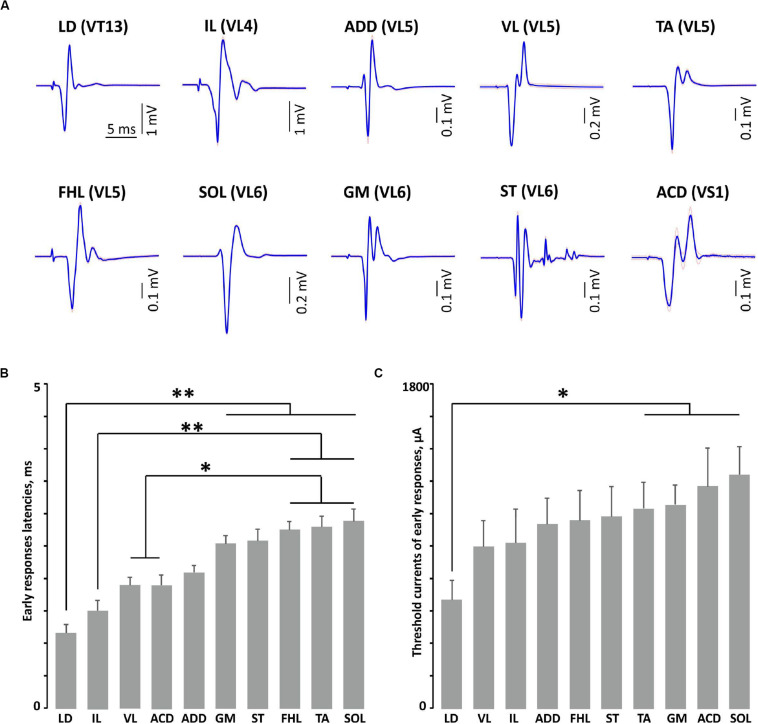
The examples of averaged evoked potentials (SE plotted by dotted line) **(A)** and the latencies **(B)** and threshold currents **(C)** of early responses of mm. longissimus dorsi (LD), abductor caudae dorsalis (ACD), iliacus (IL), adductor magnus (ADD), vastus lateralis (VL), semitendinosus (ST), tibialis anterior (TA), gastrocnemius medialis (GM), soleus (SOL), and flexor hallucis longus (FHL) at maximal or submaximal current at “optimal” stimulation. **p < 0.01, *p < 0.05.

Transvertebral SCS over the VT12–VS1 vertebrae (Figure 1) of non-anesthetized decerebrated rats evoked site-specific EMG patterns of activity in the tested muscles. Examples of evoked potential dynamics with increasing current are presented in Figure 2. The sensory-evoked potentials (namely, MR on Figure 2), similar to the motor ones (ER, on Figure 2), appear at lower currents in response to the first impulse, but the sensory-evoked potentials are absent after the second impulse. The motor-evoked potentials increase similarly with further current increases in response to the first and to the second impulses, whereas the sensory-evoked potentials decay. Thus, the motor and sensory responses illustrated classical recruiting dynamics; the MR (H-wave) was suppressed by the ER (M-wave) as the amplitude of the stimulation increased (Hoffman, 1910; Gerasimenko et al., 2006). We also observed the late reflex component in some muscles, but this was not consistent (Figure 3A, ST). The latencies of ERs were almost equal to each other in all individual responses at one stimulation point, indicating reproducibility of the recorded motor-evoked potentials and the stability of the experimental model (Figures 2, 3).

**FIGURE 4:**
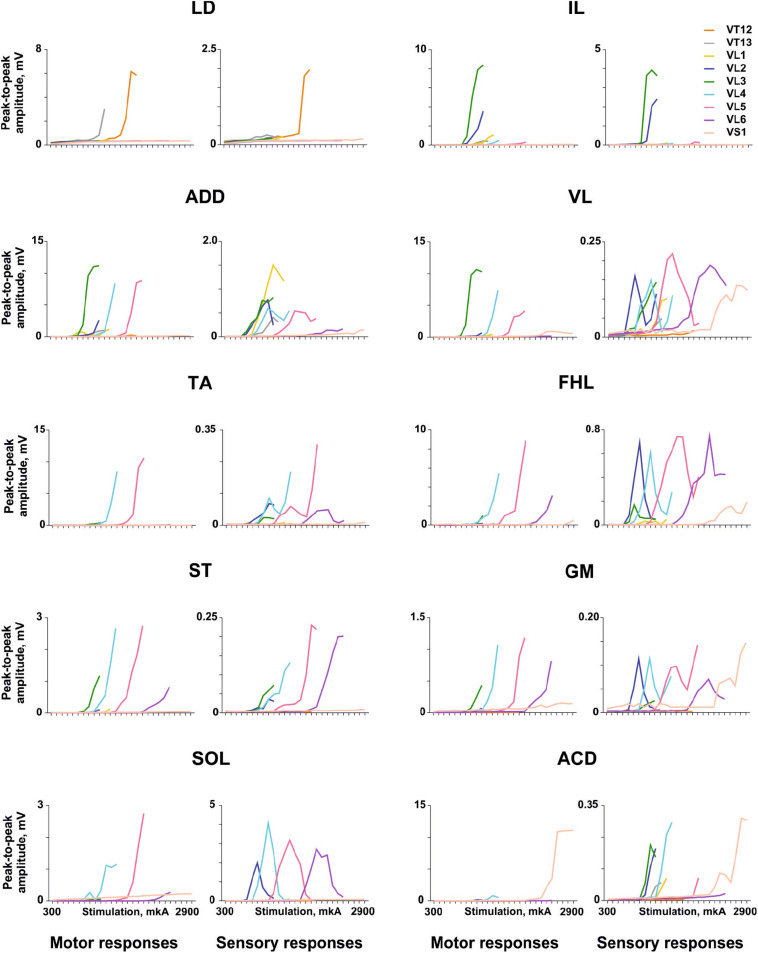
Examples of the recruitment curves for mm. longissimus dorsi (LD), abductor caudae dorsalis (ACD), iliacus (IL), adductor magnus (ADD), vastus lateralis (VL), semitendinosus (ST), tibialis anterior (TA), gastrocnemius medialis (GM), soleus (SOL), and flexor hallucis longus (FHL) plotted for motor and sensory responses in the same animal when the electrical stimulation was delivered at the VT12–VS1 vertebrae.

The examples of recruitment curves for individual muscles plotted for motor (early) and sensory responses of the same animal when the electrical stimulation was delivered at the VT12–VS1 vertebrae are presented in Figure 4. *For motor responses*, the saturation of peak-to-peak amplitudes was observed for some muscles (LD, VL, and ACD). For the different muscles, the “optimal” vertebrae where the stimulation causes a maximal recruitment curve slope were clearly different. For example, the set of LD recruitment curves had a maximum slope at the VT12 stimulation, the set of ACD recruitment curves had a maximum slope at the VS1 stimulation, and so on. When the vertebrae adjacent to the “optimal” one were stimulated, the recruitment curves had slopes close to the maximal value.

*For sensory responses*, the recruitment curves frequently had a typical inverted U-shape (e.g., FHL, SOL, GM, and VL) due to depression of the sensory responses by motor ones. Their threshold current was lower than the threshold current of the motor responses, as the sensory responses appear to arise due to activation of more excitable dorsal roots that are more closely situated to the stimulation electrode. The number of vertebrae where the stimulation caused a suprathreshold sensory response was greater than the number of vertebrae where the stimulation caused a motor response, in good agreement with previous findings (Roy et al., 2012).

**FIGURE 5:**
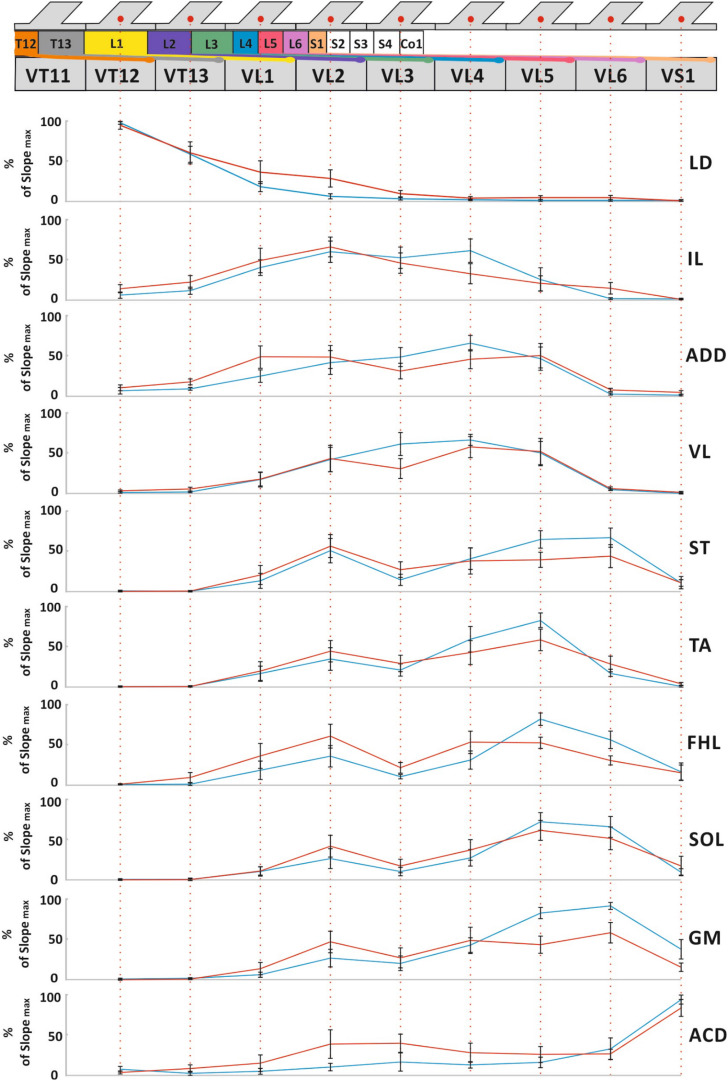
The mean skeletotopy of spinal cord segments in relation to the VT12–VS1 vertebrae (the T11–S1 segments, their roots, and DRGs are marked by different colors corresponding to Figure 2) versus the averaged distributions of normalized slopes for mm. longissimus dorsi (LD), abductor caudae dorsalis (ACD), iliacus (IL), adductor magnus (ADD), vastus lateralis (VL), semitendinosus (ST), tibialis anterior (TA), gastrocnemius medialis (GM), soleus (SOL), and flexor hallucis longus (FHL) at the stimulation of these vertebrae. Motor responses (blue), sensory responses (red). The length of all vertebrae presented are equalized for simplicity.

The averaged distributions of the normalized slopes versus the mean skeletotopy of the VT12–VS1 region for motor and sensory responses are presented in Figure 5. The significance of the difference between each pair of distributions of *motor responses* is presented in Table 1. They are subdivided into five groups. The distribution for LD has a maximum at the VT12 stimulation; the distributions for IL, ADD, and VL have an inverted U-shape with a flat maximum at the VL2–VL4 stimulation (IL) or at VL4 (ADD and VL); the distributions for TA, FHL, and ST have maxima at the VL5 stimulation and additional submaxima at the VL2 stimulation; the distributions for SOL and GM have maxima at the VL6 stimulation and additional submaxima at the VL2 stimulation; and the distribution for ACD has a maximum at the VS1 stimulation. Notably, the VL1 and VL3 vertebrae are not optimal for any of the 10 muscles under consideration.

**TABLE 1:** The significance of the difference between distributions of slopes of motor responses (χ^2^ two-sample Bonferroni-adjusted test, the significant differences on the *p* < 0.05 level are in boldface).

	**IL**	**ADD**	**TA**	**FHL**	**VL**	**ST**	**GM**	**SOL**	**ACD**
LD	**126.9**	**132.5**	**158.5**	**155.9**	**155.3**	**160.3**	**169.1**	**164.3**	**151.3**
IL		5.4	**35.9**	**61.6**	13.8	**54.6**	**79.4**	**73.6**	**98.2**
ADD			20.4	**48.8**	5.6	**44.7**	**63.9**	**58.5**	**93.3**
TA				19.8	15.2	19.1	**34.2**	**24.2**	**88.9**
FHL					**45.8**	3.9	8.7	3.4	**58.1**
VL						**39.9**	**57.4**	**52.7**	**95.4**
ST							10.6	4.2	**64.0**
GM								6.7	**42.2**
SOL									**65.0**

The distributions of *sensory responses* are wider than the distributions of motor responses. The significance of the difference between each pair of distributions is presented in Table 2. They can be subdivided into four less pronounced groups. The distribution for LD has a maximum at the VT12 stimulation; the distribution for IL has a maximum at VL2 stimulation; the distributions for ADD, VL, TA, FHL, ST, GM, and SOL have a complex form; and the distribution for ACD has maximum at the VS1 stimulation.

**TABLE 2:** The significance of the difference between distributions of slopes of sensory responses (χ^2^ two-sample Bonferroni-adjusted test, the significant differences on the *p* < 0.05 level are in boldface).

	**IL**	**ADD**	**TA**	**FHL**	**VL**	**ST**	**GM**	**SOL**	**ACD**
LD	**60.2**	**72.5**	**113.9**	**96.6**	**102.3**	**112.0**	**120.3**	**124.0**	**108.0**
IL		9.7	**31.9**	**23.2**	**23.8**	**31.6**	**42.5**	**51.7**	**45.3**
ADD			20.3	12.6	9.9	**26.7**	**34.3**	**37.4**	**44.0**
TA				8.5	11.5	5.2	8.2	9.0	**41.5**
FHL					14.4	7.1	10.9	12.4	**30.1**
VL						**22.6**	**26.7**	**29.7**	**47.2**
ST							2.5	5.9	**32.4**
GM								3.0	**31.3**
SOL									**34.1**

The distributions indirectly reflect the rostrocaudal distribution of the motoneuron pools in the rat spinal cord (Nicolopoulos-Stournaras and Iles, 1983; Mohan et al., 2015; Wenger et al., 2016). The S1 spinal cord segment is located in the VL2 vertebra due to spinal cord “ascension.” Thus, the stimulation of the VL3–VS1 vertebrae does not affect the spinal cord itself. By contrast, the stimulation of the VL2 vertebra supposedly also activates the spinal cord, leading to the additional maxima in the distribution of the sensory and motor responses mentioned above.

The examples of averaged evoked potentials of all considered muscles at the maximal or submaximal current at the “optimal” stimulation are presented in Figure 3A. Again, the “optimal” stimulation of various muscles was carried out from different vertebrae. Most of the presented evoked potentials contain the high amplitude ER followed by MR of lower amplitude. The ER latencies in all the considered muscles at the maximal current applied to “optimal” stimulation sites are presented in Figure 3B. The LD latency is significantly lower than the latencies of GM, ST, FHL, TA, and SOL (*p* < 0.01); this corresponds to the LD anatomical location and the shorter motor axon path to this muscle. Following the same logic, the latencies are significantly lower for the proximal limb muscles (IL, VL, and ACD) than for the distal ones (FHL, TA, and SOL) (IL, *p* < 0.01; VL, ACD, *p* < 0.05). The SOL is the slow muscle; the rate of rise is lower for its action potential than for those of the extensor muscles (Albuquerque and Thesleff, 1968); the spectrum of its activity covers a region of lower frequencies than do the spectra of the fast muscles (Hodson-Tole and Wakeling, 2010). This is probably why the latency is higher for SOL responses than for GM responses. The ER threshold currents increased rostrocaudally (Figures 3C, 4). The threshold current was significantly lower for LD that had optimal VT12 and VT13 stimulation vertebra (*p* < 0.05) than for TA, GM, SOL, and ACD that had optimal VL5, VL6, and VS1 stimulation vertebrae.

### Chronic Experiments

**FIGURE 6:**
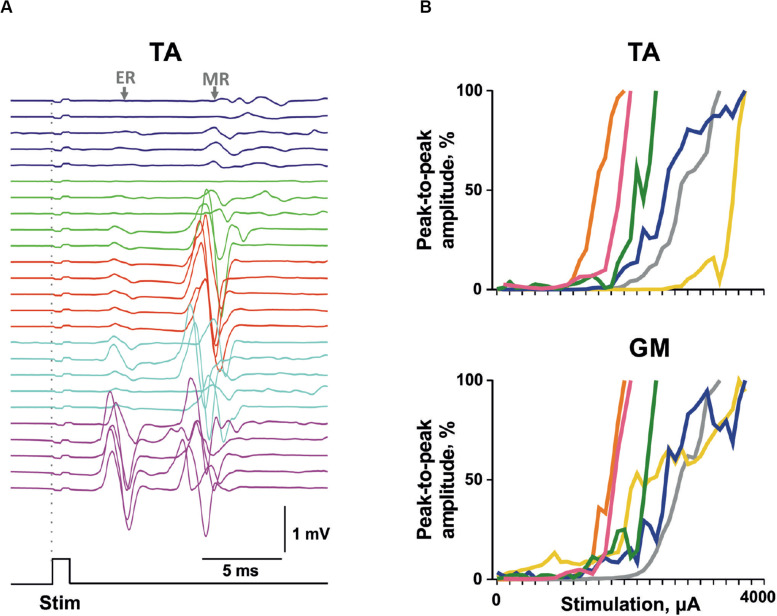
The motor-evoked potentials triggered by transvertebral stimulation in chronic animals. **(A)** Example of evoked potential recruitment dynamics with increasing current (1,600–2,000 μA) for m. Tibialis anterior (TA). **(B)** The individual recruitment curves for mm. TA and gastrocnemius medialis (GM) of different chronic rats. Stim, stimulation impulse; ER, early response; MR, medium response.

The validity of the proposed approach of electrode implantation and the possibility of causing muscle responses to vertebral stimulation in chronic conditions were checked in a group of awake animals that had survived 1 week after all the implantation surgery. The typical pattern of the evoked response of the TA muscle is presented in Figure 6A. Similar to the acute experiments, the sensory responses were decaying and the motor responses were increasing as the current increased. The recruitment curves plotted for the motor responses of the TA and GM of individual animals to the VL2 single-pulse stimulation are presented in Figure 6B. Presumably, the range of stimulation currents depends on the implantation peculiarities (e.g., variations in individual reactions to the electrodes as a foreign object, expressed by surrounding the wire with connective tissue, or a slightly different position of the wire inside the vertebral hole), whereas the slopes of the recruitment curves of different animals are rather similar.

### Comparison of the Selectivity of Transcutaneous and Transvertebral Stimulation

**FIGURE 7:**
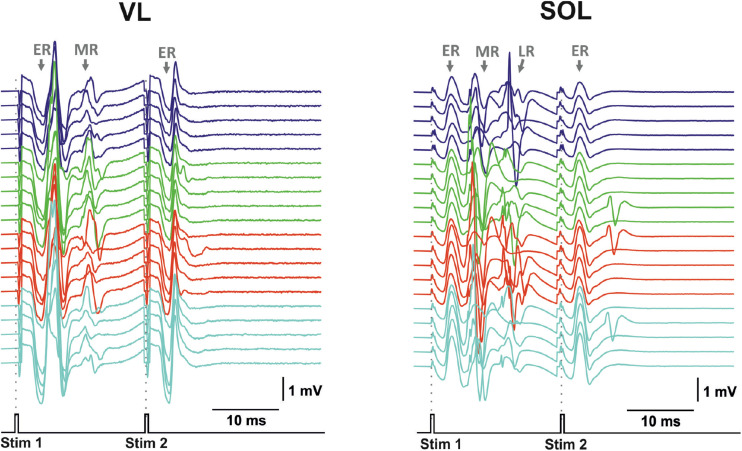
Examples of evoked potential dynamics with increasing current for transcutaneous double-pulse electrical stimulation (3,400–3,700 μA) delivered at the zone between VL2 and VL3 vertebra for mm. vastus longus (VL) and soleus (SOL). Stim, stimulation impulse; ER, early response; MR, medium response; LR, late response.

The transcutaneous and transvertebral stimulation was compared at three stimulation points located on the edges and in the center of the zone of interest: the VT12, VL2, and VL6 vertebrae. The muscle responses to double-pulse transcutaneous stimulation were qualitatively similar to those of transvertebral stimulation (Figure 7). The motor-evoked potentials were elicited in response to both stimulation pulses, whereas the sensory-evoked potentials were elicited only in response to the first one. The sensory-evoked potentials decayed with the current increase. The motor responses were chosen for comparison of the selectivity, since their distributions were narrower than those of the sensory responses (Figure 5).

**FIGURE 8:**
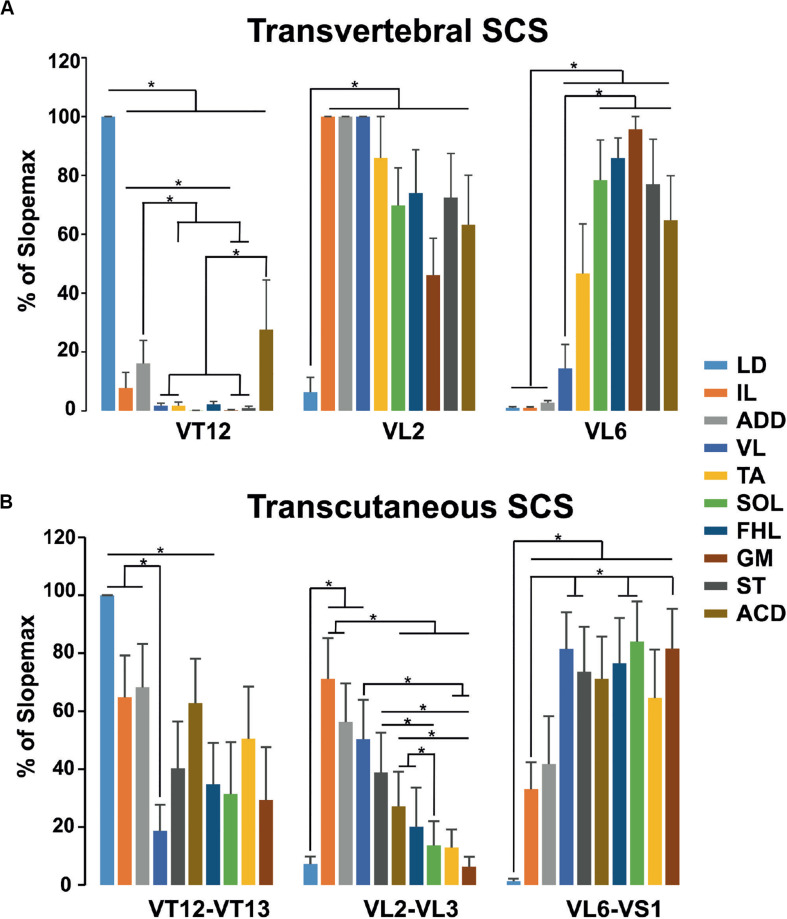
The relative slopes of recruitment curves in **(A)** transvertebral stimulation of VT12, VL2, and VL6 and **(B)** transcutaneous stimulation of VT12–VT13, VL2–VL3, and VL6–VS1 zones of mm. longissimus dorsi (LD), abductor caudae dorsalis (ACD), iliacus (IL), adductor magnus (ADD), vastus lateralis (VL), semitendinosus (ST), tibialis anterior (TA), gastrocnemius medialis (GM), soleus (SOL), and flexor hallucis longus (FHL).

The pattern of the relative slopes for transvertebral stimulation is presented in Figure 8A. The VT12 stimulation activated the LD to a maximal degree and the VL, TA, SOL, FHL, GM, and ST to a minimal degree. The relative slopes of IL, ADD, and ACD were small but significantly higher than zero. The VL2 stimulation activated the LD to a minimal degree, whereas the differences between the relative slopes of other muscles were insignificant. The VL6 stimulation revealed a significant difference between the small relative slopes of LD, IL, ADD, and VL and the large relative slopes of TA, SOL, FHL, GM, ST, and ACD. Thus, the transvertebral stimulation of VT12 and VL6 allowed the selective stimulation of different muscle groups.

The pattern of relative slopes for transcutaneous stimulation (Figure 8B) had much in common with the transvertebral stimulation, although it had some peculiarities. The selectivity of stimulation of the cutaneous zones between VT12 and VT13 and between VL6 and VS1 was lower than the selectivity of transvertebral VT12 and VL6 stimulation, respectively. On the contrary, the stimulation of the cutaneous zone between VL2 and VL3 allowed selective recruitment of the muscles having more rostrally located motoneuronal pools.

## Discussion

In the present work, we performed detailed and thorough testing of the transvertebral stimulation of thoracic (VT12–VT13), lumbar (VL1–VL6), and sacral (VS1) vertebrae to recruit the motor-evoked potentials in 10 different muscles of the trunk and hindlimbs that participate in locomotion and postural activity. This electrophysiological mapping demonstrated that the transvertebral SCS, similar to the transcutaneous SCS, has substantially specific effects on the rostrocaudally distributed sensorimotor network of the lumbar and sacral spinal segments. These effects are mainly driven by stimulation of the roots carrying sensory and motor spinal pathways.

### Site-Specific Recruitment of the Sensorimotor Pathways by Transvertebral Spinal Cord Stimulation

The spinal cord consists of rostrocaudally distributed neuronal pathways and cell groups. In particular, the lumbosacral spinal cord contains the motor pools of the trunk and hindlimb muscles (Romanes, 1951; Nicolopoulos-Stournaras and Iles, 1983; Vanderhorst and Holstege, 1997; Takahashi et al., 2010; Mohan et al., 2014, 2015), related interneuron populations (Carr et al., 1995; Dai et al., 2005; Merkulyeva et al., 2018), sensory inputs of different modalities from somatotopic regions (Rivero-Melián and Grant, 1990; Takahashi et al., 2003, 2006, 2007), and neuronal axons (Tani et al., 1994; Takahashi et al., 2010) that conduct motor commands to the musculoskeletal system during locomotor activity and postural tasks. Our aim in the present study was to use the method of transvertebral SCS to recruit specific neuronal pathways located under the thoracic, lumbar, and sacral vertebrae.

We chose ACD and LD (implanted near the VT13 vertebra) muscles as the reference points because they have the most caudal and rostral motor neuron pool localizations of all implanted muscles. In accordance, the ACD was found to be recruited at the most caudal stimulation point, namely VS1. Its motoneurons are distributed in the L5–Co1 segments with the maximum in the S1 segment (Grossman et al., 1982), where motoneurons of the hindlimb muscles are absent. The LD has multiple every-segment innervation by lateral branches of the dorsal rami of the lumbar spinal nerves (Brink and Pfaff, 1980). However, for the epaxial and hypaxial muscles, each segment of the spinal cord innervates a site located caudally; for example, a section of this muscle at the level of the VL5 vertebra is innervated by motor neurons located in the L2 and L3 segments (Takahashi et al., 2010) and the same segments receive its sensory inputs (Takahashi et al., 2003). Therefore, it makes sense that the LD implanted near the VT13 vertebra was recruited with a maximum slope during stimulation of the rostral VT12 vertebra.

A number of studies of the hindlimb motor neuron pools in various species, particularly in rats (Nicolopoulos-Stournaras and Iles, 1983; Mohan et al., 2015; Wenger et al., 2016), mice (Mohan et al., 2014), and cats (Romanes, 1951; Vanderhorst and Holstege, 1997), have shown that motor neurons innervating the proximal muscles are located more rostrally than are motor neurons innervating the distal muscles. A similar distribution pattern is well known and characteristic of the motor neurons innervating the muscles of the forelimb (McKenna et al., 2000). The proximodistal order of muscle activation has been shown in epidural (Lavrov et al., 2015) and transcutaneous (Roy et al., 2012; Gerasimenko Y. et al., 2015) stimulation experiments as the stimulation electrode moves from a rostral to a caudal direction.

However, some exceptions to this rule exist; for example, the neuronal pool for TA is rostral to the ones for GM and SOL although all these muscles are located on the shin and act to the ankle joint as a flexor and extensor, respectively (Nicolopoulos-Stournaras and Iles, 1983; Mohan et al., 2015; Wenger et al., 2016). A similar shift in motoneuron pools can be observed for the vastus and hamstrings groups (Watson et al., 2009; Mohan et al., 2015; Wenger et al., 2016) or for the forelimb biceps and triceps motoneurons (McKenna et al., 2000; Greiner et al., 2020). This may reflect the embryonic and phylogenetic origin of the TA and quadriceps group from the dorsal muscle mass and the origin of the SOL, GM, and hamstring group from the ventral one (Diogo et al., 2016).

The different maps of rat motoneuronal pools diverged in detail (Nicolopoulos-Stournaras and Iles, 1983 vs. Mohan et al., 2015 vs. Wenger et al., 2016). Furthermore, the response of a muscle to stimulation may depend indirectly on the location of its motoneuronal pool (due to root anastomosis, the peculiarities of current distribution over vertebrae, and so on). For example, Borrell et al. (2017) found a significant but partial coincidence of motoneuronal pools with intraspinal microstimulation-evoked movement patterns. This is why the following computational procedure was developed for each muscle. Initially, we calculated the maximal peak-to-peak amplitudes for sensory and motor responses at each stimulation point. We then constructed the recruitment curves based on those values and chose the recruitment curve with the maximal slope.

One of the outcomes of this study was the generation of maps of muscle-evoked potentials calculated on the basis of the averaged distributions of the normalized slopes of the recruitment curves. Our results are generally in good agreement with the data on the motoneuronal pool distribution, since the pattern of normalized slopes for proximal muscles has peaks in more rostral segments than for distal ones. The method of mapping is sufficiently sensitive even to reveal the abovementioned flexor–extensor shift of the motor neuron pool distribution. The aggregated map of the hindlimb muscles obtained is located more caudally than previously described (Nicolopoulos-Stournaras and Iles, 1983; Mohan et al., 2015) and is more similar to the data presented by Wenger et al. (2016); this requires further evaluation.

### Neuroanatomical Mechanisms of the Transvertebral Spinal Cord Stimulation

The effects of epidural and transcutaneous stimulation on the hindlimb muscles are similar to each other in that they depend on the site of stimulation in a similar manner in the rat (Capogrosso et al., 2013), cat (Musienko et al., 2013), and human (Zhu et al., 1998; Minassian et al., 2007; Roy et al., 2012; Sayenko et al., 2015; Hofstoetter et al., 2018). The H-wave appears primarily upon stimulation of the upper lumbar segments of the spinal cord or the vertebrae over them (Roy et al., 2012; Sayenko et al., 2015; Hofstoetter et al., 2018), since the dorsal root input zones into these segments are accessible for the electrical current first. The excitability is higher in the Ia afferents in the dorsal roots responsible for H-wave (Erlanger and Gasser, 1937; Lloyd and Chang, 1948) than in the low-threshold efferents in the ventral roots inducing the M-wave. The dorsal roots are anatomically much closer to the electrode and separated from the ventral roots due to the relatively large diameter of the spinal cord. The M-wave, however, may emerge with a further significant current increase. This sequence of wave appearance is also confirmed by simulation (Danner et al., 2011).

In the lower lumbar spinal cord, the dorsal and ventral roots are closer to each other (Figure 1B). In this case, the threshold currents of the M- and H-waves could be closer. Both the M- and H-waves can occur simultaneously with increasing current (Capogrosso et al., 2013). Similarly, in the lumbar vertebrae, the dorsal and ventral roots of the lower lumbar segments are located close to each other, especially in the areas near the intervertebral foramina that lead to the early appearance of the M-wave. However, in these vertebrae, the spinal cord in rats (Gelderd and Chopin, 1977; our data Figure 5), in cats (Shkorbatova et al., 2019), and in humans (Barson, 1970) may actually be absent. The M-wave may appear together with the H-wave (Zhu et al., 1998; Roy et al., 2012; Musienko et al., 2013) or at a slightly higher stimulation magnitude (Minassian et al., 2007).

The transvertebral SCS used in our work is similar to the transcutaneous one by its effects on spinal sensorimotor pathways. Firstly, the distributions are wider for the sensory-evoked potentials than for the motor ones, as was shown for transcutaneous stimulation of the human spinal cord (Roy et al., 2012). Potentially, the sensory responses may be elicited by stimulation of dorsal roots passing closer to dorsally located stimulating electrodes over the spinal cord. Therewith, the sensory pathways in the stimulating roots have wide projections and collateralization (Redett et al., 2005) while coming and switching at the spinal level. The motor responses are elicited more specifically by stimulation of the ventral roots of the segment, presumably, in their exit from the vertebral canal below the corresponding vertebra (Danner et al., 2011). Every segmental group of ventral root contains (with some individual variations) the particular set of motor axons running from motoneuron pools specific for the segment. Secondly, the responses to both types of stimulation indirectly reflect the rostrocaudal distributions of motoneuronal pools; that is, LD, IL, and ADD are active to a greater degree during the stimulation of the more rostral point (vertebrae VT12 or VT12–VT13 cutaneous zone), whereas SOL, FHL, and GM are active to a greater degree during the stimulation of the more caudal point (vertebrae VL6 or VL6–VS1 cutaneous zone). Therefore, taking into account a more superficial and distant location from the spinal canal and larger area of electrodes, the transcutaneous stimulation would appear to capture a wider zone of spinal pathways relative to the transvertebral stimulation.

Transvertebral intraoperative stimulation through the pedicle screws is widely used to monitor possible root trauma and the quality of screw implantation (Calancie et al., 1992, 1994; Toleikis et al., 2000; Danesh-Clough et al., 2001). This technique is qualitatively similar to the one used in our work, except that it is lateralized, whereas we placed the electrode at the midline in the center of the spinous process. This stimulation in sheep (Danesh-Clough et al., 2001), pig (Lenke et al., 1995), and humans (Calancie et al., 1994) showed that the amplitude and latency of the EMG response in different muscles depend on the stimulated vertebra, again reflecting the rostrocaudal distribution of motoneuronal pools in the spinal cord. The EMG data presented previously (Calancie et al., 1994; Chansakul and Nair, 2012) allow us to suppose that, with this type of stimulation, the M-wave may appear already in the near threshold current.

The low values of all latencies obtained when the “optimal” vertebra is stimulated (Figure 3B) indicate the involvement of the ventral roots, resulting in the M-wave. The different latencies of the M-waves in different muscles are associated with the length of the motoneuron axons that innervate the particular muscle: the more proximal the muscle is in relation to the spinal cord, the shorter is its M-wave latency, and *vice versa*. The M-waves predominantly appeared in the threshold current in transvertebral stimulation. Interestingly, the early appearance of the M-wave was also observed by Pavlova et al. (2019) during deep subcutaneous stimulation of the intervertebral area. Presumably, the threshold currents for excitation of motor axons and Ia/Ib fibers are similar for stimulation of the caudal segments of the spinal cord (Capogrosso et al., 2013). In the case of SCS through pedicle screws, the threshold currents rise as the distance between the screw and the neural structures increases (Montes et al., 2012). The differences in threshold currents observed in our work (Figure 3C) may be associated with anatomical differences in the structure of the rat vertebrae. Supposedly, the rostrocaudal increase of the rat’s spinous process height, calculated as Vd-VBd-SCd following Jaumard et al. (2015), leads to an increase in the distance from the stimulation point to the stimulated structures of the spinal cord.

### The Relevance of the Spinal Pathways Neuromodulation by Transvertebral Spinal Cord Stimulation

Although there are limitations in using a decerebrate preparation, our results show that transvertebral SCS can be further used in acute and chronic experiments on intact and injured animal models to access spinal pathways, such as the locomotor or visceral networks, and to investigate the neuronal control of the sensorimotor and autonomic functions. The relationship between spinal and vertebral levels are rather variable, especially for the more caudal segments (Needles, 1935; Shkorbatova et al., 2019). That is why the precise level for epidural SCS can only be well determined after a thorough neuroanatomical dissection and histology.

The important advantage of transvertebral stimulation is that it mostly affects the roots emerging from/entering into the spinal canal of the corresponding vertebra and containing sensory and motor fibers to the homologous segment. This has a special value when stimulating more caudal vertebrae where the cauda equina is formed by roots from ascending lumbosacral segments. The data obtained confirm that the transvertebral and transcutaneous stimulation approaches are selective in acting on the individual roots forming the cauda equina at the specific level from which they depart. Simply counting the vertebrae provides rather objective information about targeting the spinal cord region during the *in vivo* stage of the experiment.

One problem of transcutaneous stimulation is the difficulty in fixation of the stimulating electrode on the skin, as it is easily movable in the rat and, especially, in unanesthetized, freely behaving animals in chronic experiments. Moreover, animals have cutaneous trunk muscles that cover the back and sides of the animal body. Though the muscle is innervated from C7–T1 spinal segments (Theriault and Diamond, 1988), it responds to the stimulation of the dorsal aspects of the trunk skin by flicking or puckering the skin (Petruska et al., 2014). This causes movement of the cutaneously fixed sticky electrode during the stimulation. We also cannot exclude the direct influence of the electrical current, which may cause this muscle to contract. Possibly, for these reasons, the cutaneous stimulation was used in chronic rats in experiments where the exact position of the stimulation does not make much sense. For example, for management of neuropathic pain, relatively large (45 mm × 5 mm) adhesive electrodes were used, and they were repositioned on the dorsal rami of spinal nerves L1–L6 before the stimulation session (Somers and Clemente, 2006). However, this approach is not appropriate for more thorough examinations of the influence of stimulation points on the peculiarities of the muscle response or of locomotion.

The transcutaneous SCS may modulate corticospinal excitability and improve functional outcomes of rehabilitation (Powell et al., 2016). Possibly, the transvertebral SCS may be more selective than the transcutaneous one. There are clinical protocols using transvertebral stimulation intended to assess the functional state of spinal tracts and nerve roots after the operations that lead to potential risk of spinal cord damage, for example a simulation with the needle placed in the spinous process (Komanetsky et al., 1998; Wilson-Holden et al., 2000) or through pedicle screws (Calancie et al., 1992, 1994; Toleikis et al., 2000; Danesh-Clough et al., 2001). The latter allows an intraoperative monitoring of EMG activity of different muscle groups of human legs. This activity topically depends on the stimulation level (Chansakul and Nair, 2012). Meanwhile, the screws may be used for neuromodulation in further treatment procedures (Edidin, 2017). In addition to neurophysiological testing, the transvertebral SCS can be studied as a neurorehabilitation method in paralyzed animal models (Courtine et al., 2009) and in patients with vertebro-spinal pathology (Gill et al., 2018; Wagner et al., 2018), as it is much simpler to apply, less invasive, and safer compared with epidural SCS. The value of the proposed approach to trigger rostrocaudally distributed spinal pathways is a crucial feature for neuromodulation treatments (Wenger et al., 2014, 2016).

Further experiments on chronic rats with severe SCI and daily stimulating sessions, independent of Ichiyama et al. (2005), Lavrov et al. (2006) or in combination with modulating pharmacological agents (Ichiyama et al., 2008; Musienko et al., 2011; Moshonkina et al., 2016), will test the clinical relevance of transvertebral SCS and the advisability of its translation to practical medicine.

## Data Availability Statement

The raw data supporting the conclusions of this article will be made available by the authors, without undue reservation.

## Ethics Statement

The animal study was reviewed and approved by Ethics Commission of the Pavlov Institute of Physiology.

## Author Contributions

PS, VL, NP, and PM conceived the experiments. PS, VL, and PM wrote the manuscript. PS, VL, DK, AP, and PM edited the manuscript. PS, VL, OG, NP, DK, AP, and EB performed the research. PS and VL analyzed the data. MP supervised the study. All authors contributed to the article and approved the submitted version.

## Conflict of Interest

The authors declare that the research was conducted in the absence of any commercial or financial relationships that could be construed as a potential conflict of interest.

## References

[B1] Albuquerque E. X. Thesleff S. (1968). A comparative study of membrane properties of innervated and chronically denervated fast and slow skeletal muscles of the rat. *Acta Physiol. Scand.* 73 471–480. 10.1111/j.1365-201X.1968.tb10886.x 5708174

[B2] Barson A. J. (1970). The vertebral level of termination of the spinal cord during normal and abnormal development. *J. Anat.* 106 489–497. 5423940 PMC1233424

[B3] Borrell J. A. Frost S. Peterson J. Nudo R. J. (2017). A three-dimensional map of the hindlimb motor representation in the lumbar spinal cord in Sprague-Dawley rats. *J. Neural Eng.* 14:016007 10.1088/1741-2552/14/1/016007 PMC5270506 27934789

[B4] Brink E. E. Pfaff D. W. (1980). Vertebral muscles of the back and tail of the albino rat (*Rattus norvegicus* albinus). *Brain Behav. Evol.* 17 1–47. 10.1159/000121788 7370723

[B5] Calancie B. Lebwohl N. Madsen P. Klose K. J. (1992). Intraoperative evoked EMG monitoring in an animal model. A new technique for evaluating pedicle screw placement. *Spine* 17 1229–1235. 10.1097/00007632-199210000-00017 1440014

[B6] Calancie B. Madsen P. Lebwohl N. (1994). Stimulus-evoked EMG monitoring during transpedicular lumbosacral spine instrumentation, Initial clinical results. *Spine* 19 2780–2786. 10.1097/00007632-199412150-00008 7899979

[B7] Capogrosso M. Gandar J. Greiner N. Moraud E. M. Wenger N. Shkorbatova P. (2018a). Advantages of soft subdural implants for the delivery of electrochemical neuromodulation therapies to the spinal cord. *J. Neural Eng.* 15:026024. 10.1088/1741-2552/aaa87a 29339580

[B8] Capogrosso M. Wagner F. B. Gandar J. Moraud E. M. Wenger N. Milekovic T. (2018b). Configuration of electrical spinal cord stimulation through real-time processing of gait kinematics. *Nat. Protoc.* 13 2031–2061. 10.1038/s41596-018-0030-9 30190556

[B9] Capogrosso M. Wenger N. Raspopovic S. Musienko P. Beauparlant J. Luciani L. B. (2013). A computational model for epidural electrical stimulation of spinal sensorimotor circuits. *J. Neurosci.* 33 19326–19340. 10.1523/JNEUROSCI.1688-13.2013 24305828 PMC6618777

[B10] Carr P. A. Huang A. Noga B. R. Jordan L. M. (1995). Cytochemical characteristics of cat spinal neurons activated during fictive locomotion. *Brain Res. Bull.* 37 213–218. 10.1016/0361-9230(94)00271-2 7541702

[B11] Chansakul C. Nair D. R. (2012). “Evoked potential monitoring,” in *Anesthesia for Spinal Surgery*, ed. Farag E. (Cambridge: Cambridge University Press), 10.1017/CBO9780511793851.009

[B12] Courtine G. Gerasimenko Y. van den Brand R. Yew A. Musienko P. Zhong H. (2009). Transformation of nonfunctional spinal circuits into functional states after the loss of brain input. *Nat. Neurosci.* 12 1333–1342. 10.1038/nn.2401 19767747 PMC2828944

[B13] Dai X. Douglas J. R. Jordan L. M. (2005). Localization of spinal neurons activated during locomotion using the c-fos immunohistochemical method. *J. Neurophysiol.* 93 3442–3452. 10.1152/jn.00578.2004 15634712

[B14] Danesh-Clough T. Taylor P. Hodgson B. Walton M. (2001). The use of evoked EMG in detecting misplaced thoracolumbar pedicle screws. *Spine* 26 1313–1316. 10.1097/00007632-200106150-00008 11426144

[B15] Danner S. M. Hofstoetter U. S. Ladenbauer J. Rattay F. Minassian K. (2011). Can the human lumbar posterior columns be stimulated by transcutaneous spinal cord stimulation? A modeling study. *Artif. Organs.* 35 257–262. 10.1111/j.1525-1594.2011.01213.x 21401670 PMC4217151

[B16] Diogo R. Bello-Hellegouarch G. Kohlsdorf T. Esteve-Altava B. Molnar J. L. (2016). Comparative myology and evolution of marsupials and other vertebrates, with notes on complexity, bauplan, and “Scala Naturae”. *Anat. Rec.* 299 1224–1255. 10.1002/ar.23390 27342702

[B17] Dobson K. L. Harris J. (2012). A detailed surgical method for mechanical decerebration of the rat. *Exp. Physiol.* 97 693–698. 10.1113/expphysiol.2012.064840 22406523

[B18] Edgerton V. R. Gerasimenko Y. Roy R. Lu D. C. (2013). The regents of the University of California, applicants. Transcutaneous spinal cord stimulation: noninvasive tool for activation of locomotor circuitry. International Patent Application WO2013/071309.

[B19] Edidin A. A. (2017). Modulating nerves within bone using bone fasteners. U.S. Patent No: 9,724,151. Washington, DC: U.S. Patent and Trademark Office.

[B20] Erlanger J. Gasser H. S. (1937). *Electrical Signs of Nervous Activity.* Philadelphia: University Pennsylvania Press.

[B21] Gad P. Kreydin E. Zhong H. Latack K. Edgerton V. R. (2018a). Non-invasive neuromodulation of spinal cord restores lower urinary tract function after paralysis. *Front. Neurosci.* 12:432. 10.3389/fnins.2018.00432 30008661 PMC6034097

[B22] Gad P. Lee S. Terrafranca N. Zhong H. Turner A. Gerasimenko Y. (2018b). Non-invasive activation of cervical spinal networks after severe paralysis. *J. Neurotrauma* 35 2145–2158. 10.1089/neu.2017.5461 29649928 PMC6119225

[B23] Gelderd J. B. Chopin S. F. (1977). The vertebral level of origin of spinal nerves in the rat. *Anat. Rec.* 188 45–48. 10.1002/ar.1091880106 869231

[B24] Gerasimenko I. P. Lavrov I. A. Bogacheva I. N. Shcherbakova N. A. Kucher V. I Musienko P. E. (2003). Features of stepping pattern formation in decerebrated cats under epidural spinal cord stimulation. *Ross Fiziol Zh Im I M Sechenova* 89 1046–1057. 14758628

[B25] Gerasimenko Y. Gorodnichev R. Moshonkina T. Sayenko D. Gad P. Edgerton V. R. (2015). Transcutaneous electrical spinal-cord stimulation in humans. *Ann. Phys. Rehabil. Med.* 58 225–231. 10.1016/j.rehab.2015.05.003 26205686 PMC5021439

[B26] Gerasimenko Y. P. Lu D. C. Modaber M. Zdunowski S. Gad P. Saenko D. (2015). Noninvasive reactivation of motor descending control after paralysis. *J. Neurotrauma* 32 1968–1980. 10.1089/neu.2015.4008 26077679 PMC4677519

[B27] Gerasimenko Y. Gorodnichev R. Puhov A. Moshonkina T. Savochin A. Selionov V. (2014). Initiation and modulation of locomotor circuitry output with multisite transcutaneous electrical stimulation of the spinal cord in non-injured humans. *J. Neurophysiol.* 113 834–842. 10.1152/jn.00609.2014 25376784

[B28] Gerasimenko Y. Kozlovskaya I. Edgerton V. R. (2016). Sensorimotor regulation of movements. *Fiziol Cheloveka* 42 106–117. 10.7868/s0131164616010094 27188153

[B29] Gerasimenko Y. P. Lavrov I. A. Courtine G. Ichiyama R. M. Dy C. J. Zhong H. (2006). Spinal cord reflexes induced by epidural spinal cord stimulation in normal awake rats. *J. Neurosci. Methods* 157 253–263. 10.1016/j.jneumeth.2006.05.004 16764937

[B30] Gill M. L. Grahn P. J. Calvert J. S. Linde M. B. Lavrov I. A. Strommen J. A. (2018). Neuromodulation of lumbosacral spinal networks enables independent stepping after complete paraplegia. *Nat. Med.* 24 1677–1682. 10.1038/s41591-018-0175-7 30250140

[B31] Greiner N. Barra B. Schiavone G. James N. Fallegger F. Borgognon S. (2020). Recruitment of upper-limb motoneurons with epidural electrical stimulation of the primate cervical spinal cord. *bioRxiv* [Preprint]. 10.1101/2020.02.17.952796 PMC7815834 33469022

[B32] Grossman M. L. Basbaum A. I. Fields H. L. (1982). Afferent and efferent connections of the rat tail flick reflex (a model used to analyze pain control mechanisms). *J. Comp. Neurol.* 206 9–16. 10.1002/cne.902060103 7096630

[B33] Harkema S. Gerasimenko Y. Hodes J. Burdick J. Angeli C. Chen Y. (2011). Effect of epidural stimulation of the lumbosacral spinal cord on voluntary movement, standing, and assisted stepping after motor complete paraplegia: a case study. *Lancet* 377 1938–1947. 10.1016/S0140-6736(11)60547-3 21601270 PMC3154251

[B34] Hodson-Tole E. F. Wakeling J. M. (2010). Variations in motor unit recruitment patterns occur within and between muscles in the running rat (*Rattus norvegicus*). *J. Exp. Biol.* 210 2333–2345. 10.1242/jeb.004457 17575038

[B35] Hoffman P. (1910). Beitrage zur Kenntnis der menschlichen Reflexe mit besonderer Berucksichtigung der elektrischen Erscheinungen. *Arch. F. Physiol.* 1 223–256.

[B36] Hofstoetter U. S. Freundl B. Binder H. Minassian K. (2018). Common neural structures activated by epidural and transcutaneous lumbar spinal cord stimulation: elicitation of posterior root-muscle reflexes. *PLoS One* 13:e0192013. 10.1371/journal.pone.0192013 29381748 PMC5790266

[B37] Ichiyama R. M. Gerasimenko Y. Jindrich D. L. Zhong H. Roy R. R. Edgerton V. R. (2008). Dose dependence of the 5-HT agonist quipazine in facilitating spinal stepping in the rat with epidural stimulation. *Neurosci. Lett.* 438 281–285. 10.1016/j.neulet.2008.04.080 18490105 PMC3598626

[B38] Ichiyama R. M. Gerasimenko Y. P. Zhong H. Roy R. R. Edgerton V. R. (2005). Hindlimb stepping movements in complete spinal rats induced by epidural spinal cord stimulation. *Neurosci. Lett.* 383 339–344. 10.1016/j.neulet.2005.04.049 15878636

[B39] Jaumard N. V. Leung J. Gokhale A. J. Guarino B. B. Welch W. C. Winkelstein B. A. (2015). Relevant anatomic and morphological measurements of the rat spine. *Spine* 40 E1084–E1092. 10.1097/BRS.0000000000001021 26731709

[B40] Karayannidou A. Zelenin P. V. Orlovsky G. N. Sirota M. G. Beloozerova I. N. Deliagina T. G. (2009). Maintenance of lateral stability during standing and walking in the cat. *J. Neurophysiol.* 101 8–19. 10.1152/jn.90934.2008 19004997 PMC2637002

[B41] Komanetsky R. M. Padberg A. M. Lenke L. G. Bridwell K. H. Russo M. H. Chapman M. P. (1998). Neurogenic motor evoked potentials: a prospective comparison of stimulation methods in spinal deformity surgery. *J. Spinal Disord.* 11 21–28. 9493766

[B42] Lavrov I. Gerasimenko Y. P. Ichiyama R. M. Courtine G. Zhong H. Roy R. R. (2006). Plasticity of spinal cord reflexes after a complete transection in adult tats: relationship to stepping ability. *J. Neurophysiol.* 96 1699–1710. 10.1152/jn.00325.2006 16823028

[B43] Lavrov I. Musienko P. E. Selionov V. A. Zdunowski S. Roy R. R. Edgerton V. R. (2015). Activation of spinal locomotor circuits in the decerebrated cat by spinal epidural and/or intraspinal electrical stimulation. *Brain Res.* 1600 84–92. 10.1016/j.brainres.2014.11.003 25446455

[B44] Lenke L. G. Padberg A. M. Russo M. H. Bridwell K. H. Gelb D. E. (1995). Triggered electromyographic threshold for accuracy of pedicle screw placement. An animal model and clinical correlation. *Spine* 20 1585–1591. 10.1097/00007632-199507150-00006 7570173

[B45] Lloyd D. P. Chang H. T. (1948). Afferent fibres in muscle nerves. *J. Neurophysiol.* 11 199–227. 10.1152/jn.1948.11.3.199 18865009

[B46] McKenna J. E. Prusky G. T. Whishaw I. Q. (2000). Cervical motoneuron topography reflects the proximodistal organization of muscles and movements of the rat forelimb: a retrograde carbocyanine dye analysis. *J. Comp. Neurol.* 419 286–296. 10.1002/(sici)1096-9861(20000410)419:3<286::aid-cne2>3.0.co;2-3 10723005

[B47] Merkulyeva N. Veshchitskii A. Gorsky O. Pavlova N. Zelenin P. V. Gerasimenko Y. (2018). Distribution of spinal neuronal networks controlling forward and backward locomotion. *J. Neurosci.* 38 4695–4707. 10.1523/JNEUROSCI.2951-17.2018 29678875 PMC5956987

[B48] Minassian K. Jilge B. Rattay F. Pinter M. M. Binder H. Gerstenbrand F. (2004). Stepping-like movements in humans with complete spinal cord injury induced by epidural stimulation of the lumbar cord: electromyographic study of compound muscle action potentials. *Spinal Cord* 42 401–416. 10.1038/sj.sc.3101615 15124000

[B49] Minassian K. Persy I. Rattay F. Dimitrijevic M. R. Hofer C. Kern H. (2007). Posterior root-muscle reflexes elicited by transcutaneous stimulation of the human lumbosacral cord. *Mus. Nerve* 35 327–336. 10.1002/mus.20700 17117411

[B50] Minev I. R. Musienko P. Hirsch A. Barraud Q. Wenger N. Moraud E. M. (2015). Biomaterials. Electronic dura mater for long-term multimodal neural interfaces. *Science* 347 159–163. 10.1126/science.1260318 25574019

[B51] Mohan R. Tosolini A. P. Morris R. (2014). Targeting the motor end plates in the mouse hindlimb gives access to a greater number of spinal cord motor neurons: an approach to maximize retrograde transport. *Neuroscience* 274 318–330. 10.1016/j.neuroscience.2014.05.045 24892760

[B52] Mohan R. Tosolini A. P. Morris R. (2015). Segmental distribution of the motor neuron columns that supply the rat hindlimb: a muscle/motor neuron tract-tracing analysis targeting the motor end plates. *Neuroscience* 307 98–108. 10.1016/j.neuroscience.2015.08.030 26304758

[B53] Montes E. De Blas G. Regidor I. Barrios C. Burgos J. Hevia E. (2012). Electromyographic thresholds after thoracic screw stimulation depend on the distance of the screw from the spinal cord and not on pedicle cortex integrity. *Spine* 12 127–132. 10.1016/j.spinee.2011.09.006 21996524

[B54] Moshonkina T. R. Shapkova E. Y. Sukhotina I. A. Emeljannikov D. V. Gerasimenko Y. P. (2016). Effect of combination of non-invasive spinal cord electrical stimulation and serotonin receptor activation in patients with chronic spinal cord lesion. *Bull. Exp. Biol. Med.* 161 749–754. 10.1007/s10517-016-3501-4 27785645

[B55] Musienko P. Heutschi J. Friedli L. van den Brand R. Courtine G. (2012). Multi-system neurorehabilitative strategies to restore motor functions following severe spinal cord injury. *Exp. Neurol.* 235 100–109. 10.1016/j.expneurol.2011.08.025 21925172

[B56] Musienko P. van den Brand R. Maerzendorfer O. Larmagnac A. Courtine G. (2009). Combinatory electrical and pharmacological neuroprosthetic interfaces to regain motor function after spinal cord injury. *IEEE Trans. Biomed. Eng.* 56 2707–2711. 10.1109/TBME.2009.2027226 19635690

[B57] Musienko P. van den Brand R. Märzendorfer O. Roy R. R. Gerasimenko Y. Edgerton V. R. (2011). Controlling specific locomotor behaviors through multidimensional monoaminergic modulation of spinal circuitries. *J. Neurosci.* 31 9264–9278. 10.1523/JNEUROSCI.5796-10.2011 21697376 PMC3422212

[B58] Musienko P. E. Bogacheva I. N. Gerasimenko I. P. (2005). Significance of peripheral feedback in stepping movement generation under epidural spinal cord stimulation. *Ross Fiziol Zh Im I M Sechenova* 91 1407–1420. 16493922

[B59] Musienko P. E. Bogacheva I. N. Savochin A. A. Kilimnik V. A. Gorskii O. V. Nikitin O. A. (2013). Non-invasive transcutaneous spinal cord stimulation facilitates locomotor activity in decerebrated and spinal cats. *Ross Fiziol Zh Im I M Sechenova* 99 917–927. 25470942

[B60] Musienko P. E. Deliagina T. G. Gerasimenko Y. P. Orlovsky G. N. Zelenin P. V. (2014). Limb and trunk mechanisms for balance control during locomotion in quadrupeds. *J. Neurosci.* 34 5704–5716. 10.1523/JNEUROSCI.4663-13.2014 24741060 PMC3988419

[B61] Needles J. H. (1935). The caudal level of termination of the spinal cord in american whites and american negroes. *Anat. Rec.* 63 417–424. 10.1002/ar.1090630409C

[B62] Nicolopoulos-Stournaras S. Iles J. F. (1983). Motor neuron columns in the lumbar spinal cord of the rat. *J. Comp. Neurol.* 217 75–85. 10.1002/cne.902170107 6875053

[B63] Pavlova N. V. Bogacheva I. N. Bazhenova E. Yu Gorsky O. V. Moshonkina T. R. Gerasimenko Y. P. (2019). Restoration of motor functions in spinal rats by electrical stimulation of the spinal cord and locomotor training. *Russia. J. Physiol.* 105 565–577. 10.1134/S086981391905008X

[B64] Peckham P. H. Knutson J. S. (2005). Functional electrical stimulation for neuromuscular applications. *Annu. Rev. Biomed. Eng.* 7 327–360. 10.1146/annurev.bioeng.6.040803.140103 16004574

[B65] Petruska J. C. Barker D. F. Garraway S. M. Trainer R. Fransen J. W. Seidman P. A. (2014). Organization of sensory input to the nociceptive-specific cutaneous trunk muscle reflex in rat, an effective experimental system for examining nociception and plasticity. *J. Compar. Neurol.* 522 1048–1071. 10.1002/cne.23461 23983104 PMC3945951

[B66] Powell E. S. Carrico C. Raithatha R. Salyers E. Ward A. Sawaki L. (2016). Transvertebral direct current stimulation paired with locomotor training in chronic spinal cord injury: a case study. *Neurorehabilitation* 38 27–35. 10.3233/NRE-151292 26889795

[B67] Press W. H. Teukolsky S. A. Vetterling W. T. Flannery B. P. (1992). *Numerical Recipes in C: the Art of Scientific Computing*, 2nd Edn, Cambrigde: Cambrigde University Press.

[B68] Redett R. Jari R. Crawford T. Chen Y. Rohde C. Brushart T. (2005). Peripheral pathways regulate motoneuron collateral dynamics. *J. Neurosci.* 25 9406–9412. 10.1523/JNEUROSCI.3105-05.2005 16221849 PMC6725704

[B69] Rivero-Melián C. Grant G. (1990). Distribution of lumbar dorsal root fibers in the lower thoracic and lumbosacral spinal cord of the rat studied with choleragenoid horseradish peroxidase conjugate. *J. Comp. Neurol.* 299 470–481. 10.1002/cne.902990407 2243162

[B70] Romanes G. L. (1951). The motor cell columns of the lumbo-sacral spinal cord of the cat. *J. Comp. Neurol.* 94 313–363. 10.1002/cne.900940209 14832391

[B71] Roy F. D. Gibson G. Stein R. B. (2012). Effect of percutaneous stimulation at different spinal levels on the activation of sensory and motor roots. *Exp. Brain Res.* 223 281–289. 10.1007/s00221-012-3258-6 22990291

[B72] Sayenko D. G. Atkinson D. A. Floyd T. C. Gorodnichev R. M. Moshonkina T. R. Harkema S. J. (2015). Effects of paired transcutaneous electrical stimulation delivered at single and dual sites over lumbosacral spinal cord. *Neurosci. Lett.* 609 229–234. 10.1016/j.neulet.2015.10.005 26453766 PMC4679579

[B73] Schmidt R. A. Bruschini H. Tanagho E. A. (1978). Feasibility of inducing micturition through chronic stimulation of sacral roots. *Urology* 12 471–477. 10.1016/0090-4295(78)90309-6 715978

[B74] Shapkova E. Y. (2004). “Spinal locomotor capability revealed by electrical stimulation of the lumbar enlargement in paraplegic patients,” in *Progress in Motor Control*, eds Latash M. Levin M. (Champaign, IL: Human Kinetics), 253–289.

[B75] Shkorbatova P. Y. Lyakhovetskii V. A. Merkulyeva N. S. Veshchitskii A. A. Bazhenova E. Y. Laurens J. (2019). Prediction algorithm of the cat spinal segments lengths and positions in relation to the vertebrae. *Anat. Rec.* 302 1628–1637. 10.1002/ar.24054 30548810 PMC6561844

[B76] Siegel M. I. (1970). The tail, locomotion and balance in mice. *Am. J Phys. Anthropol.* 33 101–102. 10.1002/ajpa.1330330113

[B77] Somers D. L. Clemente F. R. (2006). Transcutaneous electrical nerve stimulation for the management of neuropathic pain: the effects of frequency and electrode position on prevention of allodynia in a rat model of complex regional pain syndrome type II. *Phys. Ther.* 86 698–709. 10.1093/ptj/86.5.698 16649893

[B78] Takahashi Y. Aoki Y. Douya H. Ohtori S. Kazuhisa T. (2006). Projection field of primary afferent fibers innervating the ventral portion of the lumbar intervertebral disc in the spinal cord dorsal horn. *Anat. Sci. Int.* 81 92–99. 10.1111/j.1447-073X.2006.00137.x 16800293

[B79] Takahashi Y. Aoki Y. Doya H. (2007). Segmental somatotopic organization of cutaneous afferent fibers in the lumbar spinal cord dorsal horn in rats. *Anat. Sci. Int.* 82 24–30. 10.1111/j.1447-073x.2006.00164.x 17370447

[B80] Takahashi Y. Chiba T. Kurokawa M. Aoki Y. (2003). Stereoscopic structure of sensory nerve fibers in the lumbar spine and related tissues. *Spine* 28 871–880. 10.1097/01.BRS.0000058717.43888.B9 12942001

[B81] Takahashi Y. Ohtori S. Takahashi K. (2010). Somatotopic organization of lumbar muscle-innervating neurons in the ventral horn of the rat spinal cord. *J. Anat.* 216 489–495. 10.1111/j.1469-7580.2009.01203.x 20136668 PMC2849526

[B82] Tani M. Kida M. Y. Akita K. (1994). Relationship between the arrangement of motoneuron pools in the ventral horn and ramification pattern of the spinal nerve innervating trunk muscles in the cat (*Felis domestica*). *Exp. Neurol.* 128 290–300. 10.1006/exnr.1994.1139 8076672

[B83] Theriault E. Diamond J. (1988). Intrinsic organization of the rat cutaneus trunci motor nucleus. *J. Neurophysiol.* 60 463–477. 10.1152/jn.1988.60.2.463 3171638

[B84] Toleikis J. R. Skelly J. P. Carlvin A. O. Toleikis S. C. Bernard T. N. Burkus J. K. (2000). The usefulness of electrical stimulation for assessing pedicle screw placements. *J. Spinal Disord.* 13 283–289. 10.1097/00002517-200008000-00003 10941886

[B85] Vanderhorst V. G. Holstege G. (1997). Organization of lumbosacral motoneuronal cell groups innervating hindlimb, pelvic floor, and axial muscles in the cat. *J. Comp. Neurol.* 382 46–76. 10.1002/(sici)1096-9861(19970526)382:1<46::aid-cne4>3.0.co;2-k 9136811

[B86] Wagner F. B. Mignardot J. Le Goff-Mignardot C. G. Prior J. Schurch B. Rowald A. (2018). Targeted neurotechnology restores walking in humans with spinal cord injury. *Nature* 563 65–71. 10.1038/s41586-018-0649-2 30382197

[B87] Watson C. Paxinos G. Kayalioglu G. (2009). *The Spinal Cord.* Amsterdam: Elsevier.

[B88] Wenger N. Moraud E. M. Gandar J. Musienko P. Capogrosso M. Baud L. (2016). Spatiotemporal neuromodulation therapies engaging muscle synergies improve motor control after spinal cord injury. *Nat. Med.* 22 138–145. 10.1038/nm.4025 26779815 PMC5061079

[B89] Wenger N. Moraud E. M. Raspopovic S. Bonizzato M. Di Giovanna J. Musienko P. (2014). Closed-loop neuromodulation of spinal sensorimotor circuits controls refined locomotion after complete spinal cord injury. *Sci. Transl. Med*. 6:255ra133. 10.1126/scitranslmed.3008325 25253676

[B90] Wilson-Holden T. J. Padberg A. M. Parkinson J. D. Bridwell K. H. Lenke L. G. Bassett G. S. (2000). A prospective comparison of neurogenic mixed evoked potential stimulation methods: utility of epidural elicitation during posterior spinal surgery. *Spine* 25 2364–2371. 10.1097/00007632-200009150-00016 10984790

[B91] Zhong H. Zhu C. Minegishi Y. Richter F. Zdunowski S. Roy R. R. (2019). Epidural spinal cord stimulation improves motor function in rats with chemically induced parkinsonism. *Neurorehabil. Neural Repair.* 33 1029–1039. 10.1177/1545968319876891 31684831 PMC6920580

[B92] Zhu Y. Starr A. Haldeman S. Chu J. K. Sugerman R. A. (1998). Soleus H-reflex to S1 nerve root stimulation. *Electroencephalogr. Clin. Neurophysiol.* 109 10–14. 10.1016/s0924-980x(97)00058-51 11003059

